# Speciation of Tellurium(VI) in Aqueous Solutions: Identification of Trinuclear Tellurates by ^17^O, ^123^Te, and ^125^Te NMR Spectroscopy

**DOI:** 10.3390/molecules27248654

**Published:** 2022-12-07

**Authors:** Alexander G. Medvedev, Oleg Yu. Savelyev, Dmitry P. Krut’ko, Alexey A. Mikhaylov, Ovadia Lev, Petr V. Prikhodchenko

**Affiliations:** 1 Kurnakov Institute of General and Inorganic Chemistry, Russian Academy of Sciences, Moscow 119991, Russia; 2 Faculty of Fundamental Medicine, M.V. Lomonosov Moscow State University, Moscow 119991, Russia; 3 Department of Chemistry, M.V. Lomonosov Moscow State University, Moscow 119991, Russia; 4 The Casali Center, The Institute of Chemistry and The Harvey M. Krueger Family Center for Nanoscience and Nanotechnology, The Hebrew University of Jerusalem, Jerusalem 9190401, Israel

**Keywords:** tellurates, NMR spectroscopy, trinuclear tellurate, spin-spin coupling, DFT computations, single-crystal X-ray diffraction

## Abstract

Tellurates have attracted the attention of researchers over the past decade due to their properties and as less toxic forms of tellurium derivatives. However, the speciation of Te(VI) in aqueous solutions has not been comprehensively studied. We present a study of the equilibrium speciation of tellurates in aqueous solutions at a wide pH range, 2.5–15 by ^17^O, ^123^Te, and ^125^Te NMR spectroscopy. The coexistence of monomeric, dimeric, and trimeric oxidotellurate species in chemical equilibrium at a wide pH range has been shown. NMR spectroscopy, DFT computations, and single-crystal X-ray diffraction studies confirmed the formation and coexistence of trimeric tellurate anions with linear and triangular structures. Two cesium tellurates, Cs_2_[Te_4_O_8_(OH)_10_] and Cs_2_[Te_2_O_4_(OH)_6_], were isolated from the solution at pH 5.5 and 9.2, respectively, and studied by single-crystal X-ray diffractometry, revealing dimeric and tetrameric tellurate anions in corresponding crystal structures.

## 1. Introduction

Over the past decade, the chemistry of telluric acid and its derivatives, tellurates, has increasingly attracted the attention of researchers [1,2,3,4,5]. Hydroxidocompounds of Te(VI) are stable in ambient conditions, soluble in water, and less toxic compared to other tellurium compounds [6]. They can be easily reduced, and therefore, they are attractive as precursors for a wide range of tellurium-based compounds and materials [7,8,9]. Moreover, tellurates are being intensively studied as promising materials with nonlinear optical properties [1,10,11,12,13,14], high proton conductivity [15,16], and ferroelectric and magnetic properties [17,18]. Tellurate-based materials benefit from high-temperature stability and optical transparency. Structural diversity allows the use of tellurates as electrode materials in metal-ion batteries [19,20]. Tellurates with perovskite structures are fundamentally interesting as oxide-ion conductors in photovoltaic applications [21].

However, most tellurates are produced by conventional hydrothermal solid-state reactions based on the fusion of the corresponding oxides in the presence of carbonate at high temperatures, controlling the composition of the final compound by loading the initial reagents. Hydrothermal synthesis also allows for obtaining tellurates of various structures and compositions. For example, tetrameric potassium K_2_[Te_4_O_8_(OH)_10_] and rubidium Rb[Te_2_O_4_(OH)_5_] tellurates with centrosymmetric tetranuclear [Te_4_O_8_(OH)_10_]^2−^ anion [12,22] and potassium tellurate K_2_[Te_3_O_8_(OH)_4_] [12] with infinite linear chains _∞_(Te_3_O_12_)^6−^ were prepared by a hydrothermal route. Interestingly, the crystallization of tellurates from aqueous solutions has not been widely used to obtain the corresponding Te(VI) compound. The hexameric tellurate K_8.5_[Te_6_O_27_H_9_]·0.5H_3_O·17H_2_O and several other alkali metal hydrogen tellurates containing binuclear anions were isolated from aqueous solution [23].

In our previous work, based on ^125^Te NMR studies, we demonstrated that the interaction of the telluric acid with alkali in aqueous solution results in the formation of tellurate anions of different nuclearity with dominant dinuclear and trinuclear species [9,24]. The formation of trinuclear tellurate anions corresponding to different ^125^Te NMR signals was also proposed [9,24,25]. However, the equilibrium of Te(VI) species in aqueous solutions with a wide pH range has not been comprehensively studied. Based on CSD [26] and ICSD [27] databases, there is no crystal structure containing trinuclear tellurate anions. Therefore, it is necessary to provide a systematic study of the equilibrium processes in aqueous solutions. This, in turn, can open up new aspects of forming a particular tellurate structure and selecting the conditions for obtaining a compound of a specific composition.

Herein we present a comprehensive study of the equilibrium in tellurate aqueous solutions by ^17^O, ^123^Te, and ^125^Te NMR spectroscopy. We also provide DFT calculations to support our assignments. Single-crystal X-ray diffraction studies of three samples obtained from cesium tellurate aqueous solutions are also presented.

## 2. Results and Discussion

The structural data of alkali metal tellurates obtained by crystallization from aqueous solutions can indirectly provide information on the structure of the tellurate anions in the initial solutions. Thus, mononuclear tellurate species in molecular or anionic form (Te(OH)_6_ and [TeO_6_H_4_]^2–^, respectively) and binuclear anions with different degrees of protonation [Te_2_O_10_H_4+x_]^(4–x)–^, where x = 0, 1, 2, were isolated from aqueous solutions. At lower pH values, anions of higher nuclearity (hexanuclear and tetranuclear anions) are formed. Thus, it can be assumed that, depending on pH and tellurium concentration, all these forms can coexist in solution. This stipulation is supported by some crystal structures containing both mononuclear and dinuclear species (K_6_[Te_2_O_10_H_4_][TeO_6_H_4_]·12H_2_O, Cs_2_[Te_2_O_10_H_6_][Te(OH)_6_]) [23].

In the present work, aqueous cesium tellurate solutions with different pH were investigated by ^17^O, ^123^Te, and ^125^Te NMR spectroscopy. We have chosen the cesium counter-cation to provide a maximal concentration of tellurium in the resulting solution since, to the best of our knowledge, cesium tellurates have the highest solubility in water compared to the tellurates of other alkali metals. Solutions of a wide range of pH (2.5–15.2) were obtained by the interaction of telluric acid and cesium hydroxide. The tellurium concentration was 1 M, except for the solution at pH 9.2, which contained only 0.5 M tellurium.

**Scheme 1:**
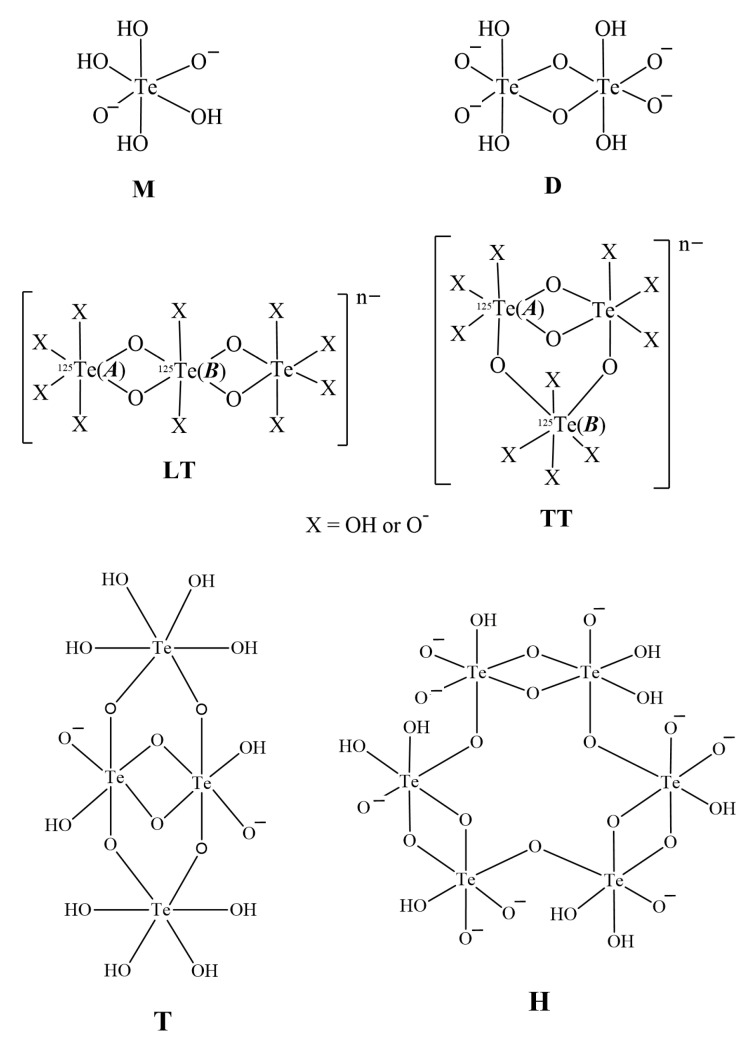
The structure of tellurate anions of different nuclearity: monomer (**M**), dimer (**D**), linear (**LT**), and triangular (**TT**) trimers, tetramer (**T**), and hexamer (**H**).

The charges of tellurate anions in solution depend on pH, and it is obvious that at high pH, the charge of the anion should be higher. Therefore, we attribute the same signals at the spectrum of different solutions to the species with the same nuclearity (monomer **M**, dimer **D**, and trimer **T**, see Scheme 1) without discussing the accurate value of the charge and degree of protonation.

### 2.1. ^125^Te NMR Studies and Solution Chemistry

**Figure 1:**
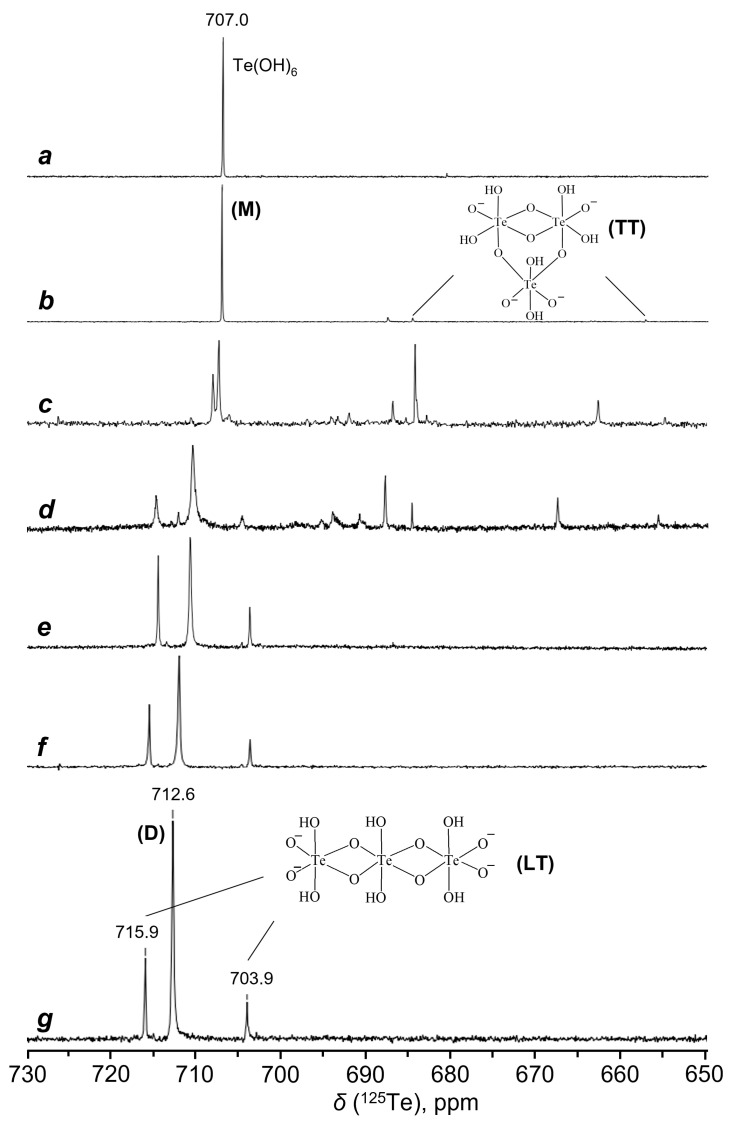
^125^Te NMR spectra of aqueous solutions with different [Te]/[Cs] ratios obtained by neutralization of telluric acid with cesium hydroxide: (**a**) solution **1** 1 M Te(OH)_6_, [Cs] = 0, pH 2.5, (**b**) solution **2** [Cs]/[Te] = 0.1, pH 5.5, (**c**) solution **3** [Cs]/[Te] = 1, pH 9.2, (**d**) solution **4** [Cs]/[Te] = 1.5, pH 11.5, (**e**) solution **5** [Cs]/[Te] = 2.5, pH 14.3, (**f**) solution **6** [Cs]/[Te] = 3.5, pH 14.8, (**g**) solution **7** [Cs]/[Te] = 4.7, pH 15.2.

The signal in ^125^Te NMR spectra with a chemical shift around 707 ppm corresponds to monomeric tellurium species (Figure 1) with different protonation degrees depending on the pH. This monomer exists as Te(OH)_6_ molecular form at low pH (<3) (Figure 1a) and in deprotonated form as [TeO_6_H_4_]^2−^ at higher pH (Figure 1b,c), which is confirmed by single-crystal X-ray data of the Te(VI) hydroxidocompounds isolated from corresponding aqueous solutions [28]. The spectrum of solution 2 with pH 5.2 contains an additional pair of signals around 685 and 658 ppm corresponding to a triangular trinuclear tellurate anion (Figure 1b). Previously, the pair of signals in the same region was incorrectly assigned to a linear trinuclear anion (see discussion below) [25].

The further increase in pH by the addition of cesium hydroxide results in the formation of a precipitate. A clear solution can be obtained only at pH 9.2, even with a decrease in the tellurium concentration from 1 to 0.5 M. The ^125^Te NMR spectra show a large number of new signals upfield from Te(OH)_6_ signal (Figure 1c,d). However, with a further increase in pH, three intense signals with chemical shift 703.9 ppm and in the intervals 710.9–712.6 ppm and 714.6–715.9 ppm remain in the spectra (Figure 1e–g). The ratio of the relative integral intensities of the first and the third signal is 1/2 and remains unchanged. At the same time, the ratio of the integral intensities of the central signal and the two lateral ones increases with an increase in pH from ~1/1 (pH 14.3) to ~3/1 (pH 15.2). The signal at 710.9–712.6 ppm apparently corresponds to tellurium atoms in dimeric anion [Te_2_O_10_H_4_]^4−^ (**D**, Scheme 1) or its protonated forms, which crystallizes from these solutions [23]. This is confirmed by the fact that the ^123^Te NMR spectrum of an aqueous solution of dimeric tellurate (see below) completely coincides with the spectra shown in Figure 1e–g. The constant ratio (1/2) of the relative integral intensities of signals at δ ~704 ppm and ~714 ppm in the ^125^Te NMR spectra at high pH values indicates that they definitely correspond to trimeric tellurate with two equivalent tellurium atoms (Figure 1e–g).

Thus, ^125^Te NMR studies of the solution showed the presence of two types of trimeric anions in solutions at different pH values. The structure of these trinuclear tellurate anions was unambiguously confirmed by additional NMR studies.

**Figure 2:**
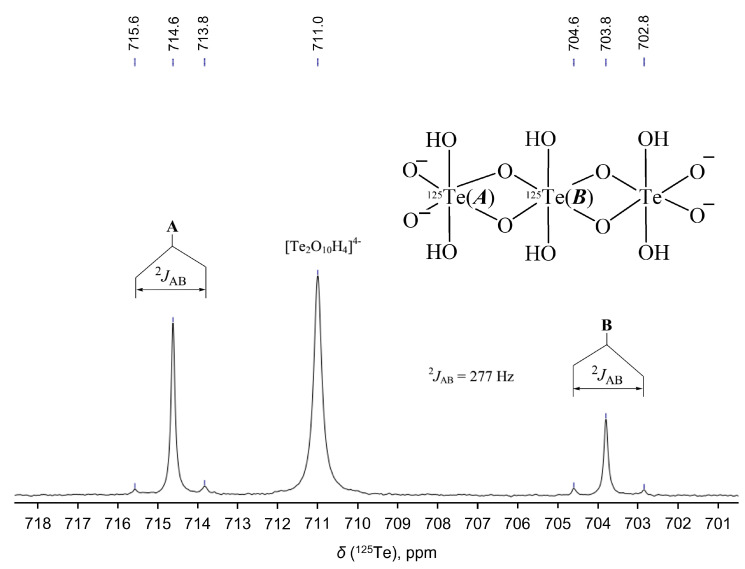
^125^Te NMR spectrum of a tellurate solution 5 ([Cs]/[Te] = 2.5, pH 14.3) (Bruker Avance-500, Bruker BioSpin GmbH, Ettlingen, Germany).

First, the ^125^Te NMR spectrum of solution **5** ([Cs]/[Te] = 2.5, pH 14.3) with a high signal-to-noise ratio was collected (Figure 2).

^125^Te satellites are visible on both signals of the trinuclear anion, which form an *AB*-spin system (^2^*J*_AB_ = 277 Hz). The relative intensity ratio of each pair of satellites to the main signal corresponds to two neighboring tellurium atoms for one of the atoms (B, δ = 703.8 ppm) and one for the other two remaining atoms (A, δ = 714.6 ppm) (Figure 2). The chemical shift difference calculated for the *AB*-spin system coincides exactly with the experimental one for main signals (10.8 ppm). The linear **LT** and triangular **TT** trimers (Scheme 1) are two possible structures that satisfy the resulting ^125^Te spectrum. Both **LT** and **TT** structures provide principally the same *AB* pattern in the ^125^Te NMR spectra.

The possible existence of the triangle trimer is confirmed by single-crystal X-ray data of tetrameric tellurates K_2_[Te_4_O_8_(OH)_10_] and Rb[Te_2_O_4_(OH)_5_] (**T**), prepared by hydrothermal route [12,22], with a similar **TT** structural fragment (Scheme 1). The same fragments were recently discovered as units of the infinite linear anionic chains in K_2_[Te_3_O_8_(OH)_4_] [12]. The hexanuclear tellurate K_8.5_[Te_6_O_27_H_9_]**·**0.5H_3_O**·**17H_2_O (**H**) is also known, in which three dimer fragments are linked by single oxygen bridges [23].

Previously, aqueous solutions of Te(OH)_6_ at low pH (<7) were studied by ^125^Te NMR spectroscopy using ^125^Te enriched (up to 92.8%) telluric acid at pH 6.78 [25]. The ^125^Te NMR spectrum, in addition to the Te(OH)_6_ signal and several minor singlets, also contains signals related to the *AB_2_* spin system with *J*_AB_ = 682.5 Hz (δ_A_ = 657.5 ppm and δ_B_ = 682.9 ppm) incorrectly assigned to the structure of linear trimeric complex **LT**.

**Figure 3:**
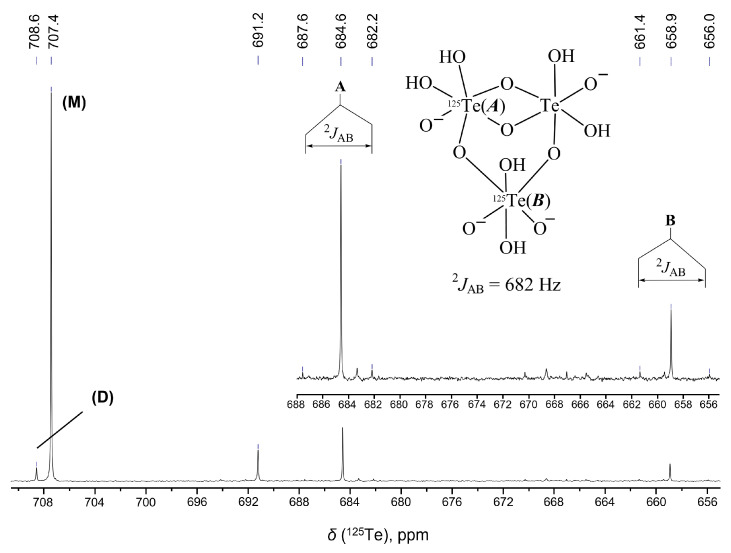
^125^Te NMR spectrum of a tellurate solution ([Te] = 0.2 M, pH 6.8).

We reproduced this experiment using the telluric acid with ^125^Te natural abundance (7.1%) (Figure 3), which is in complete agreement with the published data [25]. The ^125^Te NMR spectrum with a high signal-to-noise ratio demonstrates ^125^Te satellites from both main signals corresponding to trimeric anion. The satellites form an *AB*-spin system pattern (^2^*J*_AB_ = 682 Hz) similar to that described above.

Assignment of *AB*-spin systems depicted in Figure 2 and Figure 3 to **LT** and **TT** trimeric structures, respectively, at first glance, can be performed based on the values of spin-spin constants *^2^J*_AB_. From general considerations, ^2^*J*(^125^Te–^125^Te) values equal to 682 and 277 Hz should correspond to the interaction through two and one oxygen bridge, respectively. An unambiguous assignment of the two trimer structures can be performed by observing ^123^Te (natural abundance 0.9%) satellites from ^125^Te signals or vice versa. One set of symmetrical satellites (*AX*- and *BY*-spin systems with ^2^*J*_AX_ = ^2^*J*_BY_) should be observed for linear trimeric tellurate **LT** corresponding to the interaction through two oxygen bridges. At the same time, two sets of satellites for a signal at 714.6 ppm, corresponding to interactions through one and through two oxygen bridges, should appear with different values of ^2^*J*(^125^Te–^123^Te) for the triangular trimer **TT**.

### 2.2. ^123^Te NMR Studies

**Figure 4:**
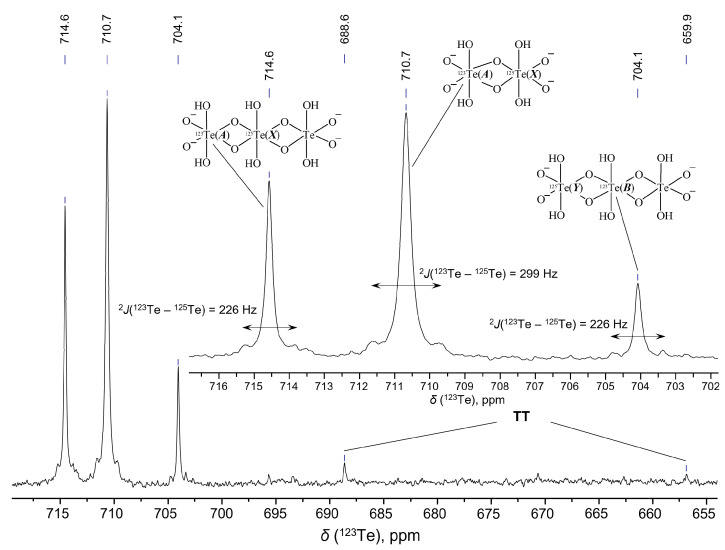
^123^Te NMR spectrum of 1 M solution of Cs_4_[Te_2_O_10_H_4_] in water (pH 14.4) (Bruker Avance-600, Bruker BioSpin GmbH, Ettlingen, Germany).

We managed to register the ^123^Te NMR spectrum with a sufficient level of signal-to-noise to observe ^125^Te satellites from ^123^Te signals at natural abundance (Figure 4). The observation of low-intensity ^123^Te satellites in the ^125^Te NMR spectrum is very difficult, taking into account the significant linewidths and the presence of additional signals from the *AB*-spin system. This explains the choice of the less-sensitive ^123^Te nucleus with a lower natural content compared to ^125^Te. A similar experiment with a natural abundance of ^123^Te was carried out to measure indirect spin-spin coupling constants *J*(^123^Te–^125^Te) in organotellurium 1,8-naphthalene derivatives [29].

In the obtained spectrum, both signals corresponding to trinuclear tellurate contain only one set of satellites ^125^Te with ^2^*J*(^123^Te–^125^Te) = 226 Hz. Multiplication by 1.206 (the ratio of resonance frequencies ***Ξ***(^125^Te)/***Ξ***(^123^Te)) gives a value of 273 Hz, which is in excellent agreement with the value of 277 Hz obtained from the ^125^Te spectrum (considering the linewidth of the signals, ~25–30 Hz).

The singlet of dimeric tellurate **D** also has one set of symmetrical ^125^Te satellites with ^2^*J*(^123^Te–^125^Te) = 299 Hz. Multiplication by 1.206 gives a value of 361 Hz, which is comparable with ^2^*J*(^125^Te–^125^Te) = 277 Hz in **LT**.

The correctness of our assignment of trimeric tellurates is confirmed by the close values of chemical shifts of the **LT** and **D** signals, which is consistent with their structural similarity. At the same time, the **TT** signals are significantly shifted upfield, especially for the Te(*B*) atom (658.9 ppm, see Figure 3). This is also confirmed by the fact that the synthesis of tetrameric tellurate **T**, which is structurally close to trimer **TT**, was carried out at pH~3 [22].

Thus, it follows from the above data that the value of ^2^*J*(^125^Te–^125^Te) in the two-bridge Te(μ-O)_2_Te fragment is approximately two times lower than in the Te(μ-O)Te fragment with one oxygen bridge. A possible explanation for this nontrivial fact is the significant difference in the Te-O-Te angles in these fragments. The values of these angles in Te(μ-O)_2_Te bridging fragments for reported structures lie in the range of 100–103° [23]. At the same time, for single bridges in the **H** hexamer, their value is ~128° [23], and for the **T** tetramer, it is ~133° [22]. An increase in the Te-O-Te angle should lead to an increase in the value of ^2^*J*(^125^Te–^125^Te). To the best of our knowledge, the values of ^2^*J*(^125^Te–^125^Te) for tellurates or related tellurium compounds are currently unknown. The dependence of the geminal coupling constant on the El-O-El bond angle was studied in detail for Sn(IV) derivatives [(R_3_Sn)_2_O] [30], formally an isoelectronic derivative of Te(VI). The data of this work confirm the fact that ^2^*J*(^119^Sn–^117^Sn) increases with increasing Sn ~ Sn angle. In the series of these compounds, a 1.5-fold increase in ^2^*J*(^119^Sn–^117^Sn) from 420.6 to 617.9 Hz is observed with an increase in the Sn-O-Sn angle from 137.1° (R = Ph) to 180.0° (R = Bn).

Dimeric tellurate **D** seems to be also present in the Te(OH)_6_ solution at pH 6.8, but at a much lower concentration (Figure 3, singlet with δ (^125^Te) = 708.6 ppm). Upfield shift with pH decrease is probably due to a change in the degree of protonation and, accordingly, the charge of the tellurate anion (obviously, protonation or deprotonation of tellurate anions cannot lead to the appearance of a new signal in the ^125^Te NMR spectrum due to fast proton exchange in aqueous solution). Low-intensity signals of trimeric tellurate **TT** at 688.6 ppm and 656.9 ppm (Figure 4) are also present in the spectrum at high pH. At the same time, the linear trimer **LT**, which, along with the **D** dimer, dominates in solutions at pH > 14, is not observed in the spectra at low pH values (Figure 1a–c and Figure 3). Probably, the remaining signals (see Figure 1c,d) can be attributed to the diverse polynuclear anionic forms of Te(VI), which exist in solutions with a relatively low [Cs]/[Te] ratio.

It should be noted that at high pH values (~14–15), the signal widths in the spectra are an order of magnitude larger (Δν_1/2_ ~ 25–40 Hz) compared to a neutral pH 6.8 solution (Δν_1/2_ ~ 4–5 Hz). This is apparently due to an increase in the rate of exchange between different tellurate anions, accompanied by the breaking of Te-O bonds in the presence of an excess of hydroxide ions. This is also confirmed by the data of ^17^O NMR spectroscopy of aqueous solutions of tellurates (see Supplementary Materials, Section S1, Figure S1).

**Scheme 2:**
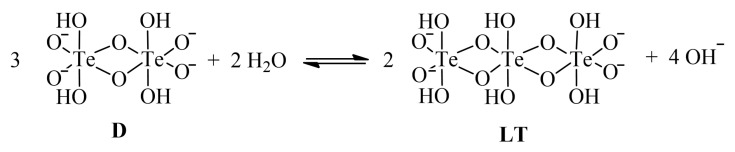
Equilibrium between dimeric and linear trimeric tellurate anions in aqueous solution.

We can conclude that an increase in the pH of tellurate solutions leads to a shift in equilibrium toward the formation of the dimeric anion **D**, which is in equilibrium with the trimeric anion **LT** (Scheme 2). The rate of interconversion between the different forms of tellurate anions in solution increases with increasing pH.

The existence of this equilibrium is confirmed by the fact that the relative content of **D** in a mixture with **LT** increases with increasing pH (see Figure 1e–g). At low pH, a number of polynuclear tellurates exist in the solution, including **TT**, whose structures apparently contain single-member oxygen bridges.

### 2.3. DFT Computations

**Figure 5:**
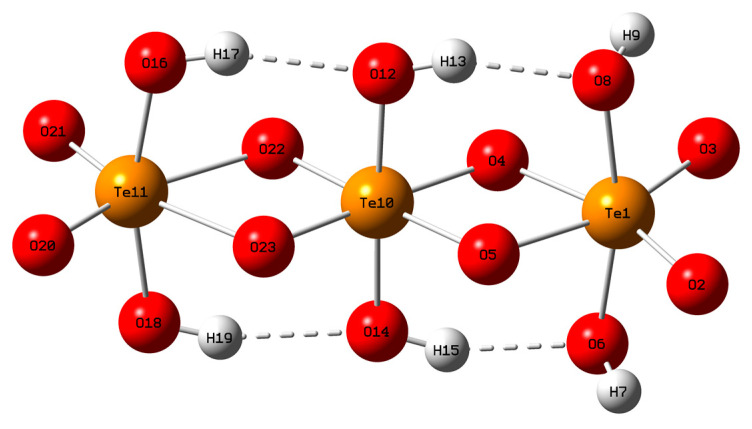
Optimized geometry of LT anion [Te_3_O_12_H_6_]^4−^. H-bonds are shown as dashed lines.

**Figure 6:**
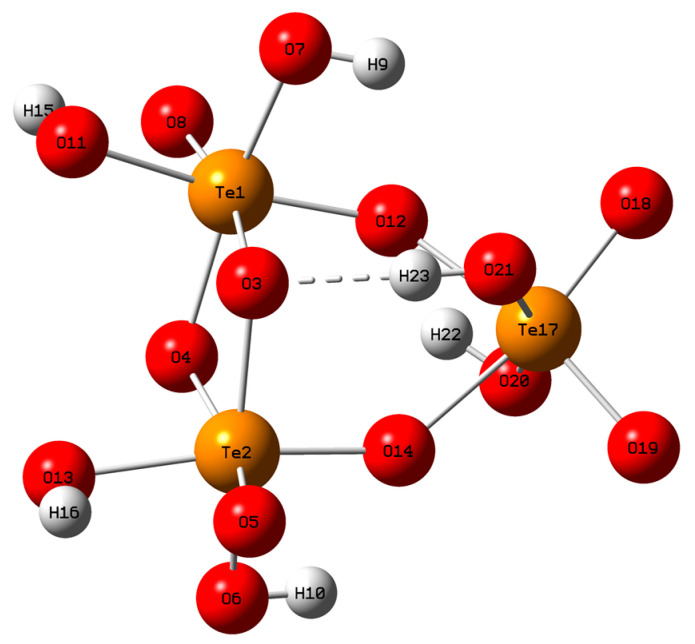
Optimized geometry of TT anion [Te_3_O_12_H_6_]^4−^. H-bond is shown as a dashed line.

In order to glean insight into the relative stability of **LT** and **TT** [Te_3_O_12_H_6_]^4−^ anions, we have conducted DFT calculations. The optimized geometries of both anions are presented in Figure 5 and Figure 6, respectively. According to these calculations, the formation of **LT** anion in the gas phase is preferable over **TT** anion (~83 kJ/mol, Supplementary Materials Tables S1 and S2). The lower energy value for the linear anion also includes the energy of the four intramolecular hydrogen bonds with O…O separations lying within 2.948–3.059 Å (Figure 5). The evaluated H-bond energies are equal to 15–20 kJ/mol (Supplementary Materials Table S3). Only one hydrogen bond is found in the structure of the triangular anion (Figure 6), with a calculated energy value of 27.9 kJ/mol. Considering the observed H-bond energies, the **LT** anion is likely to be energetically preferable (by up to ~40 kJ/mol). However, this relatively small value does not exclude the possibility of **TT** anion formation.

### 2.4. Crystal Structures

Based on the ^125^Te NMR data, solutions **2**, **3**, and **5** were selected as suitable candidates for the crystallization of trimeric tellurates. Three obtained crystals were collected from these solutions and characterized by single-crystal X-ray diffraction. Two new crystalline compounds, cesium octaoxidodecahydroxidotetratellurate Cs_2_[Te_4_O_8_(OH)_10_] **I** and cesium tetraoxidohexahydroxidoditellurate Cs_2_[Te_2_O_4_(OH)_6_] **II** were obtained from solutions **2** (pH 5.5) and **3** (pH 9.2), respectively. Previously reported cesium pentaoxidopentahydroxidoditellurate tetrahydrate Cs_3_[Te_2_O_5_(OH)_5_]**·**4H_2_O (**III**, ICSD 417438) crystals [23] were collected from solution **5**. It should be noted that the charges of tellurate anions in **II** and **III** correlate with the pH of the solution. The charge of the ditellurate anion is higher in solution **5** with pH 14.3. In addition, the tetranuclear form of tellurate **T** crystallizes from a solution with a lower pH, which corresponds to the results of the NMR studies (Figure 1).

**Figure 7:**
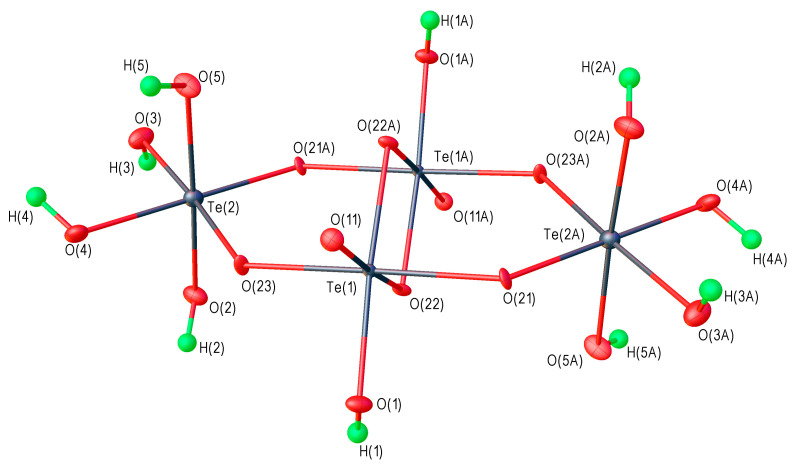
Structure of the [Te_4_O_8_(OH)_10_]^2−^ anion in compound **I**.

**Figure 8:**
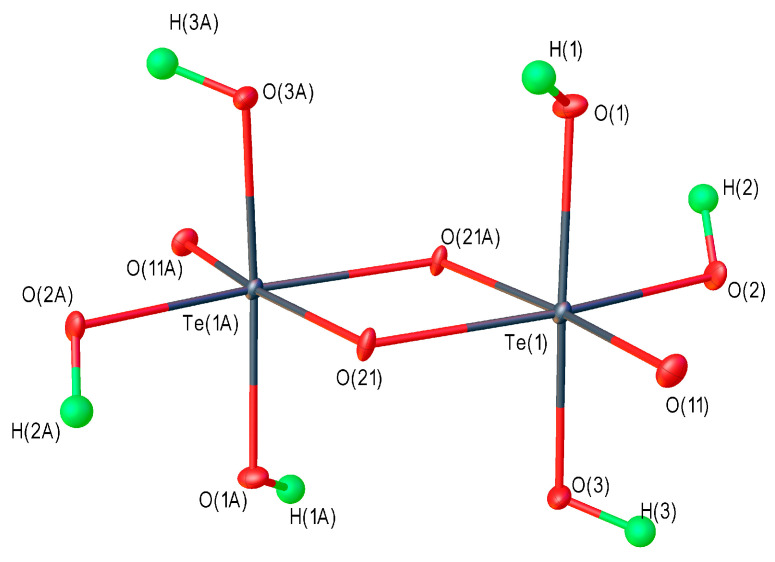
Structure of the [Te_2_O_4_(OH)_6_]^2−^ anion in compound **II**.

Both new crystalline hydrogen tellurates are ionic compounds containing centrosymmetric tetranuclear [Te_4_O_8_(OH)_10_]^2−^ and binuclear [Te_2_O_4_(OH)_6_]^2−^ anions in **I** and **II**, respectively, and Cs^+^ cations coordinated to tellurium-containing anions. Compound **I** is isostructural to previously published potassium analog K_2_[Te_4_O_8_(OH)_10_] [22]. The isomorphism of the potassium and cesium salts is not typical, and it is realized only because both structures contain a bulky anion. Selected bond distances and angles of the centrosymmetric tetranuclear [Te_4_O_8_(OH)_10_]^2−^ anion in crystal **I** are given in Supplementary Materials Table S4. The tetranuclear anion can be represented by one dimeric anion bridged by four axial oxygen atoms with two Te(OH)_6_ monomers (Figure 7). On the other hand, the [Te_4_O_8_(OH)_10_]^2−^ anion contains both linear and triangular trimeric fragments suggested by NMR studies. The tellurium atoms have a slightly distorted octahedral coordination. The geometric parameters of [Te_2_O_4_(OH)_6_]^2−^ anion in **II** are close to those found earlier in an adduct with telluric acid Cs_2_[Te_2_O_4_(OH)_6_][Te(OH)_6_] (Table S4) [23]. The Te atoms have a slightly distorted octahedral environment. As in the reported adduct, the four axial and two of the four equatorial oxygen atoms are protonated (Figure 8), resulting in a total charge of −2 for the anion. There are 11 and 12 oxygen atoms in the coordination environment of the cesium cation in **I** and **II** with Cs-O distances in the range 2.995(2)–3.631(7) and 3.055(2)–3.741(2) Å, respectively. All hydrogen atoms of tellurate anions in **I** and **II** are involved in hydrogen bonding (Supplementary Materials Table S5, Figure S2).

Unfortunately, we cannot isolate a sufficient amount of a single-phase product, which is confirmed by X-ray diffraction of the resulting powders. Further evaporation of solvent leads to the crystallization of different phases (See Supplementary Materials Section S2, Figure S3). Both crystalline powders contain small amounts of impurities. In this regard, we do not describe the yield of products, their vibrational spectra, or their elemental analysis.

Thus, the structural data of alkali metal tellurates obtained by natural crystallization from aqueous solutions can indirectly provide information on the structure of tellurate anions in the initial solutions. On the other hand, obtained results are consistent with NMR data.

## 3. Materials and Methods

### 3.1. Preparation of Solutions and Crystals

The solutions were obtained by neutralization of telluric acid with cesium hydroxide.

The [Cs]/[Te] ratio was changed from 0 to 4.7: solution **1**—[Cs]/[Te] = 0; solution **2**—[Cs]/[Te] = 0.1; solution **3**—[Cs]/[Te] = 1; solution **4**—[Cs]/[Te] = 1.5; solution **5**—[Cs]/[Te] = 2.5; solution **6**—[Cs]/[Te] = 3.5; solution **7**—[Cs]/[Te] = 4.7. Tellurium concentration in solutions **1**, **2**, and **4**–**7,** [Te] ≈ 1 M, in solution **3,** [Te] ≈ 0.5 M (Table 1).

Solution **1** was prepared by dissolving telluric acid in water.

Solution **2** was prepared by adding an appropriate volume of 4.7 M cesium hydroxide solution to solution **1**.

Solutions **5**–**7** were prepared by dissolving an appropriate amount of telluric acid in a 4.7 M cesium hydroxide solution.

Solutions **3** and **4** were prepared by mixing the corresponding volumes of solutions **2** and **5**. However, it was not possible to obtain transparent solutions in the [Cs]/[Te] ratio range greater than 0.1 and less than 1. In this concentration range, a viscous precipitate is formed, which is insoluble even after prolonged stirring at moderate heating. A transparent solution with the ratio [Cs]/[Te] = 1 (solution **3**) can only be obtained by lowering the tellurium concentration to 0.5 M.

The [Cs]/[Te] ratios in solutions **1**, **2**, and **5**–**7** were calculated from the initial amounts of telluric acid and cesium hydroxide. In this case, the tellurium concentration in the resulting solution was estimated approximately (by the mass of telluric acid and the volume of water or cesium hydroxide solution, without using volumetric analytical flasks). Accordingly, in solutions **3** and **4**, the [Cs]/[Te] ratio was estimated considering the approximate tellurium concentrations in solutions **2** and **5**.

The pH of prepared solutions was measured with Edge HI 2002-02 pH meter (Hanna Instruments, Vöhringen, Germany) equipped with HI 11310 electrode with automatic temperature compensation. The accuracy of measurements is equal to ±0.01 pH.

Cesium hydrogen tellurate Cs_4_[Te_2_O_10_H_4_]·8H_2_O was synthesized according to the previously published procedure [23]. Briefly, colorless crystals were obtained by dissolution of 6.31 g Te(OH)_6_ (0.0275 mol) in 15 mL of a 6.6 M cesium hydroxide solution. Yield 85.6% (12.89 g).

Crystalline cesium octaoxidodecahydroxidotetratellurate Cs_2_[Te_4_O_8_(OH)_10_] **I** and cesium tetraoxidohexahydroxidoditellurate Cs_2_[Te_2_O_4_(OH)_6_] **II** were obtained by slow evaporation of solvent from solutions **2** and **3**, respectively.

### 3.2. ^17^O, ^123^Te and ^125^Te NMR Spectroscopy

^125^Te NMR spectra were recorded on Bruker MSL-400, Bruker Avance-400, and Bruker Avance-500 (Bruker BioSpin GmbH, Ettlingen, Germany) spectrometers at resonance frequencies 126.24 and 157.81 MHz, respectively. ^123^Te NMR spectrum was recorded on a Bruker Avance-600 spectrometer at resonance frequency 157.05 MHz. ^17^O NMR spectra were recorded on a Bruker MSL-400 spectrometer at resonance frequency 54.24 MHz. Chemical shifts were measured using external references: aqueous solution of Te(OH)_6_ (707.0 ppm relative to Te(CH_3_)_6_ [31]) for ^125^Te and ^123^Te and water for ^17^O.

### 3.3. Computations

Gaussian09 (Gaussian Inc., Wallingford, CT, USA) was used in all computations [32]. Geometries of the linear and triangular trinuclear anion [Te_3_O_12_H_6_]^4−^ were optimized at the B3LYP/6-311G++(d,p) level using LANL2DZ basis set with effective core potential for Te atoms [33]. The normal-mode analysis did not provide imaginary frequencies for the considered species. The optimized cartesian coordinates of trinuclear anions are presented in Supplementary Materials Tables S1 and S2.

The energy of intermolecular H-bonds *E_HB_* in the considered crystals (Supplementary Materials Table S3) is evaluated according to ref. [34] as:

*E_HB_* [kJ mol^−1^] = 1124·*G_b_* [atomic units] (1)

where *G_b_* is the positively defined local electronic kinetic energy density at the H∙∙∙O bond critical point [35]. The Espinosa approach gives reasonable results for energies of intermolecular H-bonds and other non-covalent interactions [36,37,38,39].

### 3.4. Single-Crystal X-ray Diffraction

Experimental reflection intensity data for compounds **I** and **II** were collected on a Bruker D8 Venture diffractometer (Bruker AXS GmbH, Karlsruhe, Germany; graphite monochromatized Mo*K*_α_ radiation, λ = 0.71073 Å) using ω-scan mode at 100 K. Absorption corrections based on measurements of equivalent reflections were applied [40]. The structures were solved by direct methods and refined by full-matrix least-squares on F^2^ with anisotropic thermal parameters for all non-hydrogen atoms [41]. Hydrogen atoms were located from different Fourier syntheses and refined isotropically. Selected atom distances and bond angles are collected in Table S4. Selected crystallographic data for **I** and **II** are provided in Table 2. Atomic coordinates and anisotropic displacement parameters are given in Supplementary Materials (Tables S6–S9). CCDC 2215218 and 2215219 contain the supplementary crystallographic data for this paper. These data can be obtained free of charge via http://www.ccdc.cam.ac.uk/conts/retrieving.html (accessed on 30 November 2022) (or from the CCDC, 12 Union Road, Cambridge CB2 1EZ, U.K.; fax: +44 1223 336033; e-mail: deposit@ccdc.cam.ac.uk).

## 4. Conclusions

A comprehensive study of the equilibrium in tellurate aqueous solutions was carried out by ^125^Te, ^123^Te, and ^17^O NMR. The coexistence of monomeric, dimeric, and trimeric tellurate species in chemical equilibrium in the aqueous solutions of cesium tellurates at a wide pH range was proven. The formation and coexistence of trimeric tellurate anions with linear and triangular structures were shown for the first time based on NMR studies and DFT calculations. Three tellurates were crystallized from the studied solutions and studied by single-crystal X-ray analysis. Two of obtained crystals, cesium octaoxidodecahydroxidotetratellurate Cs_2_[Te_4_O_8_(OH)_10_] and cesium tetraoxidohexahydroxidoditellurate Cs_2_[Te_2_O_4_(OH)_6_], were isolated for the first time and shown to contain dimeric and tetrameric tellurate anions. The latter contains the triangular tellurate fragment, which was characterized by ^125,123^Te NMR in the solution.

## Figures and Tables

**Table 1:** Aqueous solutions with different pH obtained by neutralization of telluric acid with cesium hydroxide.

Solution	[Cs], M	[Te], M	pH
Solution **1**	0	1	2.5
Solution **2**	0.1	1	5.2
Solution **3**	0.5	0.5	9.2
Solution **4**	1.5	1	11.5
Solution **5**	2.5	1	14.3
Solution **6**	3.5	1	14.8
Solution **7**	4.7	1	15.2

**Table 2:** Crystal data, data collection, and refinement parameters for **I** and **II**.

Parameter	I	II
Formula	Cs_2_Te_4_O_18_H_10_	Cs_2_Te_2_O_10_H_6_
CCDC	2215219	2215218
fw	1074.30	687.07
Color, habit	Colorless, prism	Colorless, prism
Cryst size, mm	0.10 × 0.05 × 0.05	0.10 × 0.10 × 0.05
Cryst syst	Monoclinic	Triclinic
Space group	P21/c	P$\bar{1}$
*a*, Å	5.7174(3)	6.2963(3)
*b*, Å	8.4698(4)	6.3962(3)
*c*, Å	16.6536(9)	7.3552(4)
*α*, deg	90	67.507(2)
*β*, deg	97.436(2)	77.178(2)
*γ*, deg	90	65.661(1)
*V*, Å^3^	799.67(2)	248.63(2)
*Z*	2	1
*ρ*_calc_, g/cm^3^	4.462	4.589
*μ*, mm^−1^	11.803	13.116
*F*(000)	944	300
*θ* range, deg	2.47 to 30.46	3.01 to 30.48
Total no. of reflns	7894	4145
Unique reflns, *R*_int_	1833, 0.0422	1504, 0.015
Reflns. with *I* > 2σ(*I*)	1632	1429
No. of variables	124	76
*R*_1_ (*I* > 2σ(*I*))	0.0377	0.0161
w*R*_2_ (all data)	0.0849	0.0371
GoF on *F*^2^	1.130	1.117
Largest diffPeak/hole, e/Å^3^	1.881/−2.050	0.800/−1.034

## Data Availability

The data presented in this study are available on request from the corresponding authors.

## Supplementary Materials

The following supporting information can be downloaded at: https://www.mdpi.com/article/10.3390/molecules27248654/s1, Section S1: ^17^O NMR studies; Figure S1: ^17^O NMR spectra of: (**a**) telluric acid aqueous solution; (**b**) dimer D in water; (**c**) dimer D in water at 323 K; (**d**) dimer **D** in water, enriched by ^17^O; Figure S2. Crystal packing in **II**; Table S4: Selected bond lengths and angles in the structure of **I** and **II**; Table S1: Optimized cartesian coordinates of **LT** anion [Te_3_O_12_H_6_]^4−^; Table S2: Optimized cartesian coordinates of **TT** anion [Te_3_O_12_H_6_]^4−^; Table S3: Computed values of the electron density, *ρ*_b_, the local electronic kinetic energy density, *G*_b_, at the O…O critical point, the H-bond energy, *E*_HB_, evaluated using Equation (1); Table S5: Hydrogen-bond geometry in compounds **I** and **II**; Table S6: Fractional atomic coordinates and isotropic or equivalent isotropic displacement parameters (Å^2^) for **I**; Table S7: Atomic displacement parameters (Å^2^) for **I**; Table S8: Fractional atomic coordinates and isotropic or equivalent isotropic displacement parameters (Å^2^) for **II**; Table S9: Atomic displacement parameters (Å^2^) for **II**; Section S2: X-ray powder diffraction (XRD); Figure S3: X-ray powder diffractograms of cesium octaoxidodecahydroxidotetratellurate Cs_2_[Te_4_O_8_(OH)_10_] **I** (**a**) and cesium tetraoxidohexahydroxidoditellurate Cs_2_[Te_2_O_4_(OH)_6_] **II** (**b**) Calculated powder diffractograms were obtained using Mercury (CCDC) software.

## Sample Availability

Samples of the compounds **I**–**III** are available from the authors.
