# Supplementary material for: Speciation of Tellurium(VI) in Aqueous Solutions: Identification of Trinuclear Tellurates by 17O, 123Te, and 125Te NMR Spectroscopy
Source: Molecules. 2022 Dec 7;27(24):8654. doi: 10.3390/molecules27248654 (PMC9788595; doi:10.3390/molecules27248654)
Supplement: Supplementary file 1 [file molecules-27-08654-s001.zip › molecules-2022333-supplementary.pdf]

Supplementary Materials

Speciation of Tellurium(VI) in Aqueous Solutions: Identification of Trinuclear Tellurates by ^{17}O, ^{123}Te, and ^{125}Te NMR Spectroscopy

Section S1. *^{17}O NMR Studies*

In the ^{17}O NMR spectrum of aqueous solution of dimer **D** (Figure S1b), in addition to the signal corresponding to water oxygen atoms, there is a signal at 328 ppm and a group of unresolved signals in the region of 130-200 ppm which can be represented as three signals at 151, 177 and 189 ppm (see Figure S1d). Considering that the oxygen atoms of hydroxido groups in telluric acid correspond to a resonance signal at 124.5 ppm (Figure S1a), the signals of hydrogen tellurate anions in the region of 130-200 ppm can be attributed to various types of terminal hydroxido and oxido groups coordinated with the tellurium atom in **D** and **LT**, and the signal at 328 ppm - to the bridging oxygen atoms. The ratio of the number of bridging and terminal oxygen atoms in a mixture of **D** and **LT** weakly depends on their ratio and varies from 0.32 (**D**/**LT** = 1.5/1 (mol), pH 14.3) to 0.28 (**D**/**LT** = 4.5/1 (mol), pH 15.2). These values are in reasonable agreement with the integral ratio of signals at 328 ppm and 130-200 ppm equal to 0.24, measured for ^{17}O enriched sample (Figure S1d).

Full coincidence (with exception of signal to noise ratio) of the spectra of dimer **D** solution in water with a natural content of ^{17}O and enriched in this isotope (Figure S1b,d)) indicates the occurrence of reactions (with intermediate exchange rate in NMR time scale) involving hydroxide anions, accompanied by the breaking of all types of Te-O bonds. These reactions, as expected, accelerate with increasing temperature (Figure S1c).

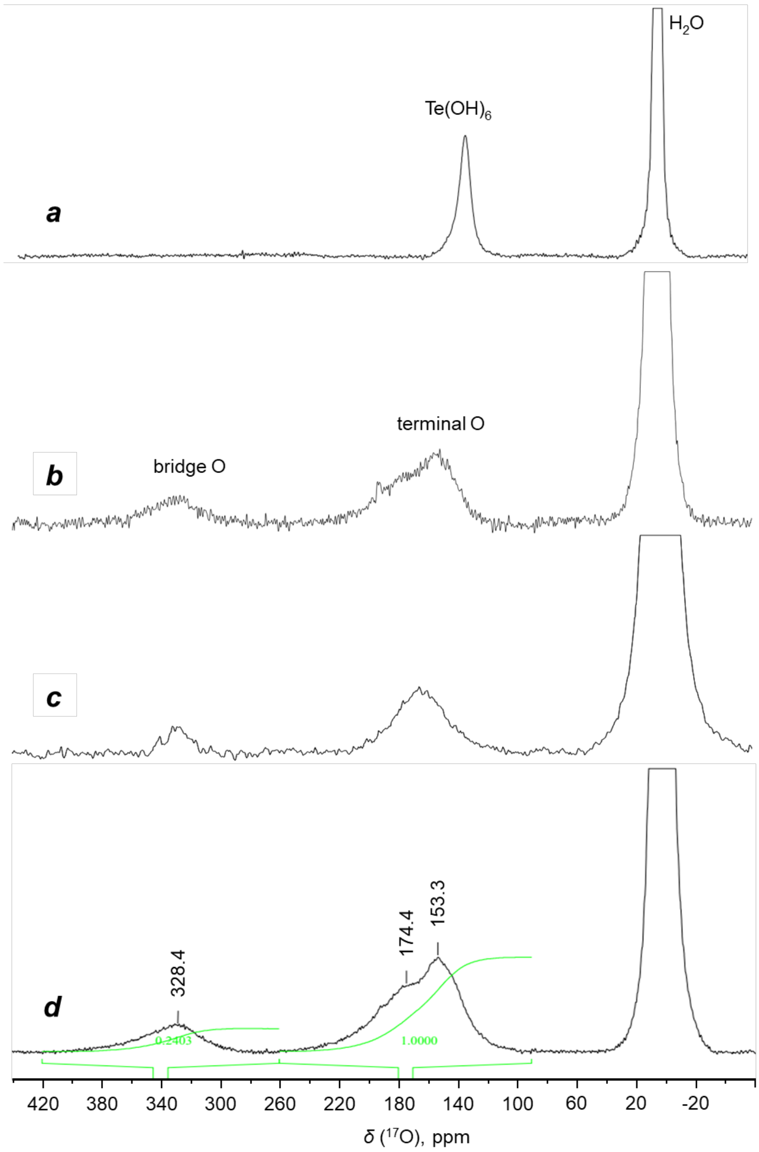

Figure S1. ^{17}O NMR spectra of: **a)** telluric acid aqueous solution; **b)** dimer **D** in water; **c**) dimer **D** in water at 323 K; **d**) dimer **D** in water, enriched by ^{17}O.

Table S1. Optimized cartesian coordinates of LT anion $[Te_3O_{12}H_6]^{4-}$.

Te	3.22330000	0.00016900	0.00017800
O	4.36083800	-0.26462400	1.45912100
O	4.35813900	0.26679700	-1.46059200
O	1.54345800	0.05962700	-1.24648600
O	1.54659100	-0.06174200	1.24457400
O	2.98443500	-2.07509900	-0.16060800
H	3.32166200	-2.32624300	0.70918400
O	2.98169000	2.07569800	0.15923300
H	3.31961300	2.32645500	-0.71039000
Te	-0.00851600	-0.00036400	0.00040200
Te	-3.30331500	0.00020900	-0.00048300
O	0.03417100	2.01134600	0.11765800
H	0.97367200	2.27139400	0.16184600
O	0.03181200	-2.01226200	-0.12171400
H	0.97096600	-2.27285700	-0.16726000
O	-3.02457000	2.03028500	0.12627400
H	-2.07125900	2.21224300	0.12267000
O	-3.02284900	-2.02977200	-0.12475100
H	-2.06917400	-2.21092700	-0.12343200
O	-4.41319300	-0.09020200	1.48437400
O	-4.41708900	0.08982600	-1.48234100
O	-1.47532300	0.08311400	-1.25856900
O	-1.46834400	-0.08309000	1.26412600

Zero-point correction= 0.108471 (Hartree/Particle)
Thermal correction to Energy= 0.131654
Thermal correction to Enthalpy= 0.132599
Thermal correction to Gibbs Free Energy= 0.057287
Sum of electronic and zero-point Energies= -1080.552839
Sum of electronic and thermal Energies= -1080.529655
Sum of electronic and thermal Enthalpies= -1080.528711
Sum of electronic and thermal Free Energies= -1080.604022

Table S2. Optimized cartesian coordinates of TT anion $[Te_3O_{12}H_6]^{4-}$.

Te	1.096216	1.606151	0.119056
Te	1.146617	-1.571836	-0.069632
O	1.067620	0.083050	-1.242978
O	1.578719	-0.042016	1.175817
O	1.217226	-2.839315	-1.441959
O	1.034943	-2.816023	1.544596
O	0.502221	2.858561	-1.371166
O	1.507816	2.859566	1.446394
H	-0.452540	2.686523	-1.368214
H	0.097434	-2.676481	1.753612
O	3.003251	1.960566	-0.591620
O	-0.791984	1.380274	0.448636
O	3.199013	-1.866561	0.011723
O	-0.764616	-1.436874	0.250933
H	3.274611	2.564760	0.113072
H	3.238727	-2.512556	-0.707626
Te	-2.381590	-0.056463	-0.034467
O	-3.531038	1.400012	-0.241925
O	-3.529115	-1.510490	-0.178830
O	-2.373164	-0.021548	2.026187
O	-1.695927	0.057099	-1.940618
H	-1.620738	0.572534	2.168045
H	-0.721848	-0.013526	-1.898145

Zero-point correction=	0.107947 (Hartree/Particle)
Thermal correction to Energy=	0.131070
Thermal correction to Enthalpy=	0.132015
Thermal correction to Gibbs Free Energy=	0.057812
Sum of electronic and zero-point Energies=	-1080.522326
Sum of electronic and thermal Energies=	-1080.499202
Sum of electronic and thermal Enthalpies=	-1080.498258
Sum of electronic and thermal Free Energies=	-1080.572461

Table S3. Computed values of the electron density, ρ_b, the local electronic kinetic energy density, G_b, at the O…O critical point, the H-bond energy, E_{HB}, evaluated using Equation (1).

H-bond [a]	Anion	d(O…O), Å	ρ_b, a.u.	G_b, a.u.	E_{HB}, kJ/mol
O16-H17…O12	LT	3.059	0.0161	0.0134	15.1
O18-H19…O14		3.055	0.0163	0.0136	15.3
O14-H15…O6		2.954	0.0228	0.01795	20.2
O12-H13…O8		2.949	0.0225	0.01769	19.9
O21-H23…O3	TT	2.850	0.0293	0.0248	27.9

[a] See Figsures 5 and 6.

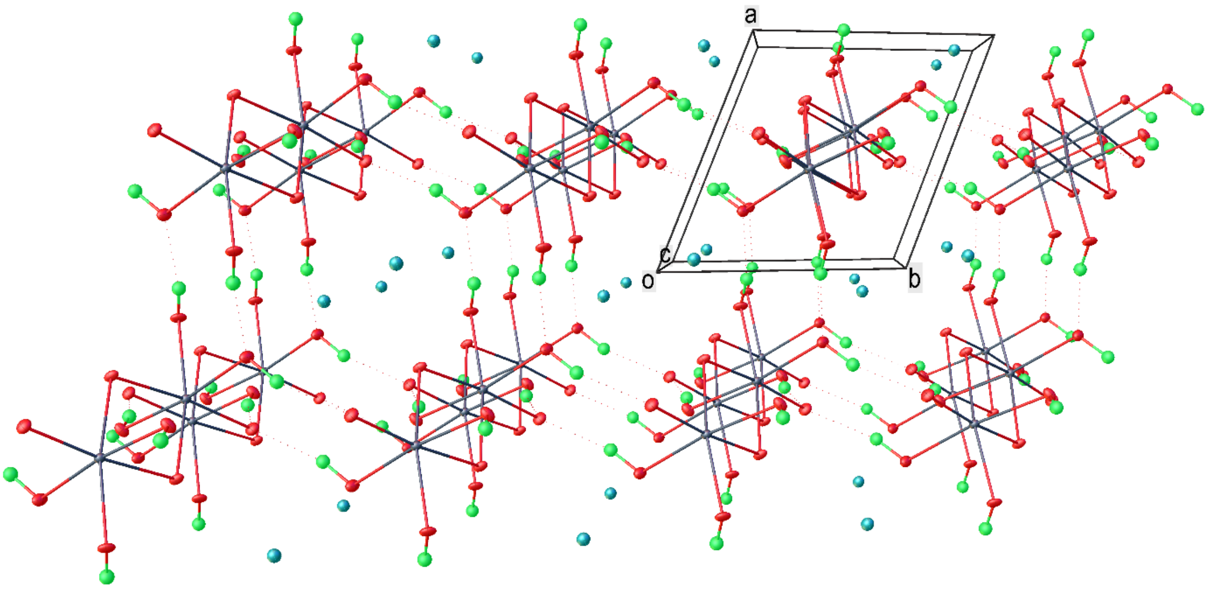

Figure S2. Crystal packing in **II.**

Table S4. Selected bond lengths and angles in the structure of **I** and **II**.

**Bond**	***d*, Å**	**Bond**	***d*, Å**
		**I**	
Te(1)-O(1)	1.885(7)	Te(2)-O(5)	1.938(7)
Te(1)-O(11)	1.833(6)	Te(2)-O(21A)	1.949(6)
Te(1)-O(21)	1.943(6)	Te(2)-O(23)	1.924(6)
Te(1)-O(22)	1.966(6)	O(1)-H(1)	0.848(10)
Te(1)-O(22A)	1.976(6)	O(2)-H(2)	0.849(10)
Te(1)-O(23)	1.960(6)	O(3)-H(3)	0.850(10)
Te(2)-O(2)	1.900(7)	O(4)-H(4)	0.850(10)
Te(2)-O(3)	1.906(7)	O(5)-H(5)	0.847(10)
Te(2)-O(4)	1.937(7)		
Angle	deg	Angle	deg
O(1)-Te(1)-O(21)	89.2(3)	O(23)-Te(1)-O(22A)	90.0(3)
O(1)-Te(1)-O(22)	90.1(3)	O(23)-Te(2)-O(21A)	97.7(3)
O(1)-Te(1)-O(22A)	169.9(3)	O(23)-Te(2)-O(4)	84.0(3)
O(1)-Te(1)-O(23)	90.5(3)	O(23)-Te(2)-O(5)	93.2(3)
O(11)-Te(1)-O(1)	94.0(3)	O(3)-Te(2)-O(21A)	87.4(3)
O(11)-Te(1)-O(21)	92.8(3)	O(3)-Te(2)-O(23)	174.5(3)
O(11)-Te(1)-O(22)	175.7(3)	O(3)-Te(2)-O(4)	90.9(3)
O(11)-Te(1)-O(22A)	96.1(3)	O(3)-Te(2)-O(5)	84.9(3)
O(11)-Te(1)-O(23)	91.7(3)	O(4)-Te(2)-O(21A)	177.8(3)
O(2)-Te(2)-O(21A)	89.0(3)	O(4)-Te(2)-O(5)	90.6(3)
O(2)-Te(2)-O(23)	90.7(3)	O(5)-Te(2)-O(21A)	90.8(3)
O(2)-Te(2)-O(3)	91.1(3)	Te(1)-O(1)-H(1)	116(4)
O(2)-Te(2)-O(4)	89.5(3)	Te(1)-O(21)-Te(2A)	131.9(3)
O(2)-Te(2)-O(5)	176.0(3)	Te(1)-O(22)-Te(1A)	100.2(3)
O(21)-Te(1)-O(22)	88.4(3)	Te(2)-O(2)-H(2)	114(4)
O(21)-Te(1)-O(22A)	89.5(3)	Te(2)-O(23)-Te(1)	131.9(3)
O(21)-Te(1)-O(23)	175.5(3)	Te(2)-O(3)-H(3)	114(4)
O(22)-Te(1)-O(22A)	79.8(3)	Te(2)-O(4)-H(4)	111(4)
O(23)-Te(1)-O(22)	87.1(3)	Te(2)-O(5)-H(5)	111(4)
		**II**	
Te(1)-O(1)	1.946(2)	Te(1)-O(21A)	1.988(2)
Te(1)-O(2)	1.924(2)	O(1)-H(1)	0.847(10)
Te(1)-O(3)	1.925(2)	O(2)-H(2)	0.845(10)
Te(1)-O(11)	1.844(2)	O(3)-H(3)	0.846(10)
Te(1)-O(21)	1.957(2)		
Angle	deg	Angle	deg
O(1)-Te(1)-O(21)	94.07(10)	O(11)-Te(1)-O(2)	97.84(10)
O(1)-Te(1)-O(21A)	87.96(10)	O(11)-Te(1)-O(3)	93.12(11)
O(2)-Te(1)-O(3)	87.86(10)	O(11)-Te(1)-O(1)	91.85(11)
O(2)-Te(1)-O(1)	86.65(10)	O(11)-Te(1)-O(21)	94.49(10)
O(2)-Te(1)-O(21)	167.62(10)	O(11)-Te(1)-O(21A)	172.45(10)
O(2)-Te(1)-O(21A)	89.69(10)	O(21)-Te(1)-O(21A)	78.00(10)
O(3)-Te(1)-O(1)	173.05(10)	Te(1)-O(1)-H(1)	115(4)
O(3)-Te(1)-O(21)	90.39(10)	Te(1)-O(2)-H(2)	109(4)
O(3)-Te(1)-O(21A)	87.76(10)	Te(1)-O(3)-H(3)	112(4)

Table S5. Hydrogen-bond geometry in compounds **I** and **II**.

H-bond	D-H, Å	H···O, Å	O···O, Å	O-H···O, °
**I**				
O(1)-H(1)···O(22)^{i}	0.85(1)	1.97(4)	2.781(9)	160(10)
O(2)-H(2)···O(5)^{ii}	0.85(1)	1.91(5)	2.714(10)	157(11)
O(3)-H(3)···O(11)^{iii}	0.85(1)	1.81(2)	2.638(10)	164(7)
O(4)-H(4)···O(11)^{iv}	0.85(1)	1.87(2)	2.696(10)	164(6)
O(5)-H(5)···O(4)^{iv}	0.85(1)	2.07(8)	2.739(10)	135(10)
**II**				
O(1)-H(1)···O(11)^{v}	0.85(1)	1.81(2)	2.642(3)	166(6)
O(2)-H(2)···O(1)^{vi}	0.85(1)	1.81(2)	2.631(3)	165(6)
O(3)-H(3)···O(11)^{vii}	0.85(1)	1.83(2)	2.668(3)	169(5)

Symmetry codes: (i) $x, -y+1/2, z+1/2$; (ii) $x-1, y, z$; (iii) $-x, -y+1, -z+1$; (iv) $-x+1, -y, -z+1$; (v) $-x+1, -y+2, -z+1$; (vi) $-x+2, -y+1, -z+1$; (vii) $-x+1, -y+1, -z+2$;.

Table S6. Fractional atomic coordinates and isotropic(*) or equivalent isotropic displacement parameters (Å^2) for **I**.

	x	*y*	*z*	U_{iso}*/U_{eq}
Cs1	−0.01887(11)	0.39779(7)	0.78522(4)	0.01165(15)
Te1	0.37346(10)	0.41505(7)	0.42711(3)	0.00411(14)
Te2	0.46585(11)	0.18768(8)	0.60727(4)	0.00892(16)
O1	0.0504(12)	0.4109(8)	0.3860(4)	0.0095(14)
H1	−0.043(8)	0.444(13)	0.418(4)	0.014*
O2	0.1803(12)	0.2443(9)	0.6470(4)	0.0121(13)
H2	0.056(6)	0.227(14)	0.614(4)	0.018*
O3	0.5999(13)	0.1189(9)	0.7122(4)	0.0140(15)
H3	0.530(15)	0.155(13)	0.750(2)	0.021*
O4	0.3386(12)	−0.0210(9)	0.5820(4)	0.0114(14)
H4	0.417(14)	−0.091(5)	0.610(5)	0.017*
O5	0.7654(12)	0.1252(9)	0.5743(4)	0.0120(13)
H5	0.749(10)	0.048(9)	0.542(6)	0.018*
O11	0.4592(12)	0.2834(8)	0.3486(4)	0.0082(13)
O21	0.4099(11)	0.6031(8)	0.3630(4)	0.0062(12)
O22	0.3046(11)	0.5531(8)	0.5160(4)	0.0061(13)
O23	0.3295(11)	0.2362(8)	0.4984(4)	0.0068(12)

Table S7. Atomic displacement parameters (Å^2) for **I**.

	U^{11}	U^{22}	U^{33}	U^{12}	U^{13}	U^{23}
Cs1	0.0126(3)	0.0119(3)	0.0101(3)	0.0016(2)	0.0003(2)	−0.0009(2)
Te1	0.0043(3)	0.0049(3)	0.0030(3)	−0.0008(2)	−0.0002(2)	−0.0001(2)
Te2	0.0086(3)	0.0106(3)	0.0074(3)	−0.0001(2)	0.0006(2)	−0.0002(2)
O1	0.006(3)	0.017(4)	0.006(3)	0.000(3)	0.001(3)	0.000(3)
O2	0.010(3)	0.015(3)	0.012(3)	−0.002(3)	0.003(3)	−0.006(3)
O3	0.015(4)	0.018(4)	0.009(3)	0.005(3)	0.004(3)	0.002(3)
O4	0.009(3)	0.012(4)	0.012(4)	0.002(3)	−0.003(3)	−0.001(3)
O5	0.014(3)	0.015(3)	0.009(3)	−0.002(3)	0.006(2)	−0.008(3)
O11	0.010(3)	0.009(3)	0.005(3)	0.000(3)	0.002(3)	−0.003(3)
O21	0.009(3)	0.006(3)	0.004(3)	−0.003(2)	0.003(2)	0.001(2)
O22	0.004(3)	0.011(3)	0.003(3)	−0.001(2)	0.000(2)	0.000(3)
O23	0.010(3)	0.007(3)	0.002(3)	−0.003(2)	−0.002(2)	0.001(2)

Table S8. Fractional atomic coordinates and isotropic(*) or equivalent isotropic displacement parameters (Å^2) for **II**.

	**x**	**y**	**z**	**Uiso*/Ueq**
Cs1	0.08290(4)	1.15530(4)	0.17975(3)	0.00914(5)
Te1	0.58092(3)	0.55687(4)	0.65158(3)	0.00456(5)
O1	0.7455(4)	0.7461(4)	0.4443(4)	0.0083(4)
H1	0.661(9)	0.890(4)	0.383(8)	0.032(15)*
O2	0.8685(4)	0.3843(4)	0.7799(3)	0.0077(4)
H2	0.982(7)	0.367(11)	0.693(6)	0.031(15)*
O3	0.4529(4)	0.3363(4)	0.8584(3)	0.0081(4)
H3	0.489(9)	0.312(10)	0.971(4)	0.026(14)*
O11	0.4495(4)	0.7940(4)	0.7701(3)	0.0080(4)
O21	0.3138(4)	0.6753(4)	0.4972(3)	0.0064(4)

Table S9. Atomic displacement parameters (Å^2) for **II**.

	U^{11}	U^{22}	U^{33}	U^{12}	U^{13}	U^{23}
Cs1	0.00886(10)	0.00832(9)	0.00985(10)	−0.00294(7)	−0.00230(7)	−0.00191(7)
Te1	0.00463(9)	0.00507(9)	0.00370(9)	−0.00104(7)	−0.00088(7)	−0.00163(7)
O1	0.0072(10)	0.0076(10)	0.0066(10)	−0.0023(9)	0.0002(8)	0.0004(8)
O2	0.0047(10)	0.0103(11)	0.0062(10)	−0.0003(8)	−0.0028(8)	−0.0021(9)
O3	0.0099(11)	0.0113(11)	0.0048(10)	−0.0066(9)	−0.0004(8)	−0.0014(9)
O11	0.0089(11)	0.0080(10)	0.0069(10)	−0.0014(9)	0.0000(8)	−0.0044(8)
O21	0.0061(10)	0.0074(10)	0.0051(10)	0.0005(8)	−0.0027(8)	−0.0035(8)

Section S2. *X-ray Powder Diffraction (XRD).*

X-ray powder diffraction measurements were performed on a D8 Advance diffractometer (Bruker AXS, Karlsruhe, Germany). XRD patterns in the range 5° to 60° 2θ were recorded at room temperature using CuK_{α} radiation under the following measurement conditions: reflection geometry, tube voltage of 40 kV, tube current of 40 mA, Ni filter, LYNXEYE detector, step scan mode with a step size 0.02° 2θ, and counting time of 0.5 s/step. XRD patterns were processed by DIFFRAC.SUITE Eva (Bruker) software. Calculated powder patterns were obtained using Mercury (CCDC) software and CIF files.

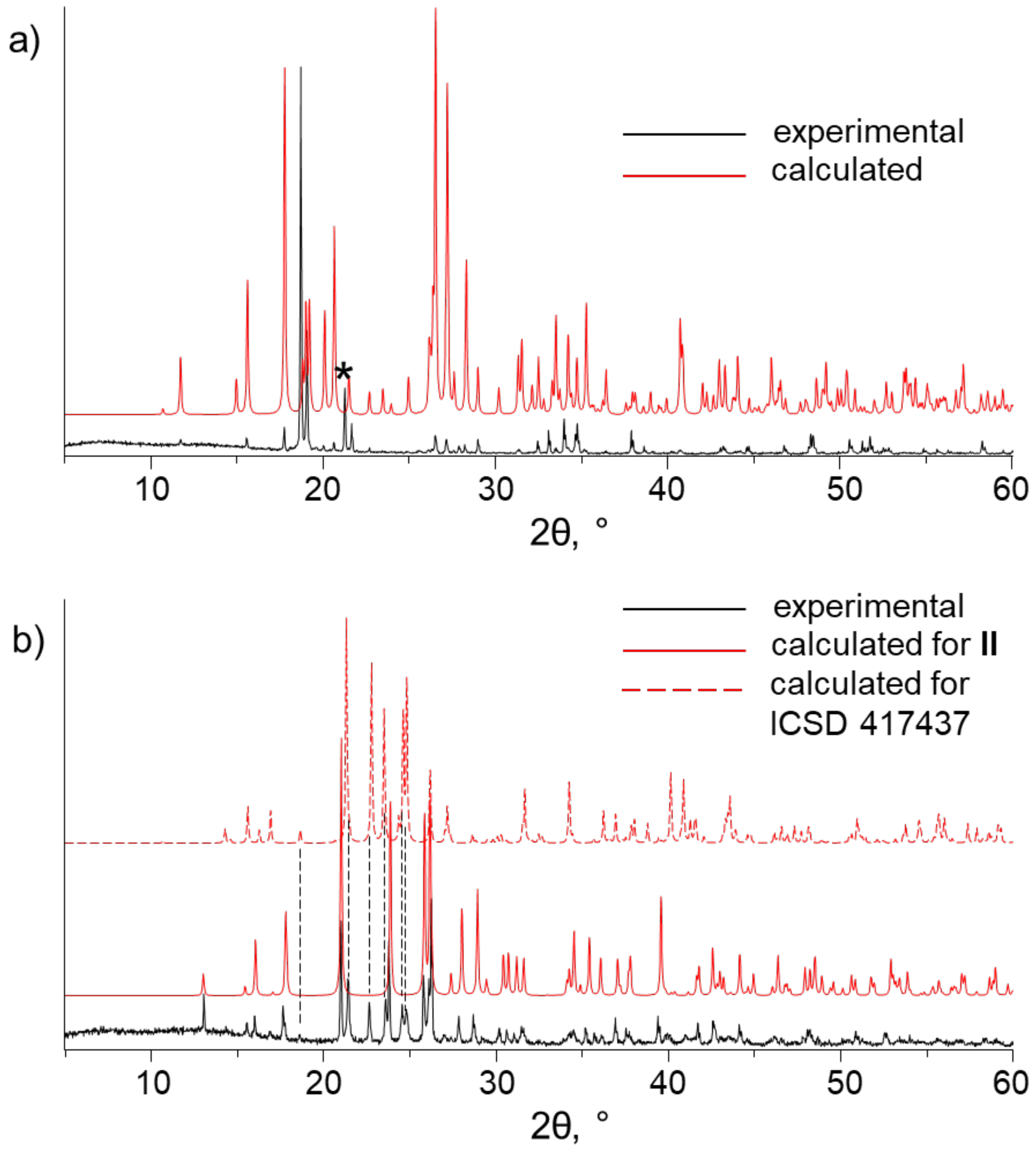

Figure S3. X-ray powder diffractograms of cesium octaoxidodecahydroxidotetratellurate $Cs_2[Te_4O_8(OH)_{10}]$ **I** (a) and cesium tetraoxidohexahydroxidoditellurate $Cs_2[Te_2O_4(OH)_6]$ **II** (b). Calculated powder diffractograms were obtained using Mercury (CCDC) software.

Experimental X-ray powder diffractograms of samples obtained from solution 2 and 3, respectively fits adequately to the calculated diffractograms obtained from single crystal data (Figure S3). Powder X-ray diffractogram of **I** contains a peak of unidentified phase at 21° (Figure S3a), while the second sample contains up to 6 additional peaks corresponding to cesium hexahydrogenditellurate telluric acid adduct $Cs_2[Te_2O_{10}H_6][Te(OH)_6]$ (ICSD 417437 [23]) impurity phase. Thus, results of the XRD studies confirm that compounds **I** and **II** are the dominant products isolated from corresponding solutions and the obtained powders contain impurities that do not allow a complete characterization of the samples.
